# Ipsilesional High Frequency Repetitive Transcranial Magnetic Stimulation Add-On Therapy Improved Diffusion Parameters of Stroke Patients with Motor Dysfunction: A Preliminary DTI Study

**DOI:** 10.1155/2016/6238575

**Published:** 2016-10-20

**Authors:** Zhiwei Guo, Yu Jin, Haitao Peng, Guoqiang Xing, Xiang Liao, Yunfeng Wang, Huaping Chen, Bin He, Morgan A. McClure, Qiwen Mu

**Affiliations:** ^1^Department of Imaging and Imaging Institute of Rehabilitation and Development of Brain Function, The Second Clinical Medical College of North Sichuan Medical College, Nanchong Central Hospital, Nanchong 637000, China; ^2^Lotus Biotech.Com LLC., John Hopkins University-MCC, Rockville, MD, USA; ^3^Department of Neurology, The Second Clinical Medical College of North Sichuan Medical College, Nanchong Central Hospital, Nanchong 637000, China; ^4^Peking University Third Hospital, Beijing, China

## Abstract

*Purpose*. The aim of this study was to evaluate the effects of high frequency repetitive transcranial magnetic stimulation (HF-rTMS) on stroke patients with motor dysfunction and to investigate the underlying neural mechanism.* Methods*. Fifteen stroke patients were assigned to the rTMS treatment (RT) group and conventional treatment (CT) group. Patients in the RT received 10 Hz rTMS stimulation on the ipsilesional primary motor cortex for 10 days plus conventional treatment of CT, which consisted of acupuncture and antiplatelet aggregation medication. Difference in fractional anisotropy (FA) between pretreatment and posttreatment and between two groups was determined. Correlations between FA values and neurological assessments were also calculated.* Results*. Both groups significantly improved the neurological function after treatment. rTMS-treated patients showed better improvement in Fugl-Meyer Assessment (FMA) score and increased FA value in motor-related white matter and gray matter cortices compared with CT-treated patients and pretreatment status. Besides, the increased FA value in the ipsilesional posterior limb of the internal capsule in RT group was significantly correlated with the improved FMA score.* Significance*. HF-rTMS could be a supplement therapy to CT in improving motor recovery in patients with stroke. And this benefit effect may be achieved through modulating the ipsilesional corticospinal tracts and motor-related gray matter cortices.

## 1. Introduction

Stroke is the major cause of adult disability worldwide. Up to 80% of the stroke patients endure motor deficits that severely lowers the quality of their daily lives [1, 2]. Most of the stroke survivors could obtain a certain degree of motor improvement after various therapies, including medication, acupuncture, movement training, and other types of interventions. So far, however, the effectiveness of these interventions remains unsatisfactory.

The neural mechanisms of motor recovery following stroke remain unknown. Recent studies indicate that the functional plasticity and structural remodeling of white microstructure could underlie the poststroke recovery process [3–6]. Transcranial magnetic stimulation (TMS) is a safe, painless, and noninvasive strategy that was first reported by Barker et al. in 1985 [7, 8]. Repetitive TMS (rTMS) is a new method that can alert activity in cortex and induce lasting effects on neuroplasticity in the cortex, and it has been used to treat depression, Parkinson's disease, stroke, and other neurological diseases in recent years. The rTMS therapy for motor recovery following stroke aims to augment neural plasticity and improve motor function based on the interhemispheric competition model, which states that inhibitory rTMS on contralesional hemisphere increases excitability in the ipsilesional motor cortex by reducing excessive interhemispheric inhibition from the contralesional motor cortex [9–11], whereas excitatory rTMS over the affected hemisphere directly increases the excitability of the ipsilesional motor cortex [11–14].

Previous plastic studies of rTMS had focused mainly on functional brain mapping. Currently, diffusion tensor imaging (DTI) has been widely used as a noninvasive tool to investigate and measure the integrity of white matter in vivo [15]. Fractional anisotropy (FA), one of DTI parameters, can quantify the degree of water diffusion and reliably visualize the microstructural status, but it is susceptible to axonal myelination as well as density and orientational coherence [16]. Reduced FA has been reported in Wallerian degeneration or destruction of white matter integrity [17] and in people with motor impairments [18]. Several DTI-based studies that evaluated the FA values along the ipsilesional corticospinal tracts (CST) suggest a decreased FA value following stroke [19–21]. After the interventions of different treatments, the progressively increased FA values in the ipsilesional CST were found positively correlated with the functional recovery of the stroke patients as measured by the Fugl-Meyer Assessment (FMA) [4, 5, 22]. Several related studies also reported that the stroke motor recovery is positively and significantly associated with the changes of FA in the contralesional hemisphere after unilateral infract [23–25]. Besides, previous low frequency rTMS studies also have shown obvious effects in improving motor function via inhibiting contralesional motor cortex and exciting the ipsilesional cortex [26, 27]. To date the research on rTMS-induced white matter modification after stroke is limited, and it is not clear if high frequency rTMS could also lead to an improvement in motor function and remodeling of white microstructures in stroke patients with motor dysfunction.

The present study aimed at determining whether high frequency rTMS-induced excitability of the ipsilesional hemisphere would cause plastic changes in the microstructure of white matter of stroke patients with motor dysfunction and the correlation with motor recovery.

## 2. Methods

### 2.1. Participants

Fifteen acute ischemic stroke patients with left hemisphere infarctions were recruited for this study from March to December 2015 at the Second Clinical Medical College of North Sichuan Medical College, Nanchong, China, with the following inclusion criteria: (1) first-ever stroke patients with a unilateral hemisphere infarct within seven days of onset; (2) the lesion being subcortical with its focal point confirmed by diffusion weighted imaging (DWI); (3) National Institutes of Health Stroke Scale (NIHSS) scores ≥2; (4) mild to moderate motor impairment lasting at least 48 hours; (5) age from 50 to 80 years; and (6) being without seizure, dysgnosia, psychosis, or other coexistent neurological/psychiatric disease. All study procedures were conducted in accordance with the Helsinki Declaration of 1975 and were approved by Institutional Review Board of the North Sichuan Medical College. Informed consent was obtained from all participants prior to enrollment into the study.

A total of 15 patients were assigned to two groups: 7 to the rTMS treatment (RT) group and 8 to the conventional treatment (CT) group. The patients in the RT group received 10 Hz rTMS treatment over the ipsilesional motor cortex for 10 days plus conventional treatment, whereas patients in CT group underwent conventional treatment including acupuncture and antiplatelet aggregation drugs medication.

### 2.2. MRI Procedure and Neurological Evaluation

Each patient received the neurological functional assessments and MRI scans two times: prior to the first rTMS session and immediately after the end of rTMS treatments (10 days of rTMS stimulation). All patients received the same medical therapy including anticoagulant (low molecular weight heparin or aspirin), brain protection (piracetam), and blood circulation protection (*Salvia miltiorrhiza*).

### 2.3. Clinical Assessment

All patients were assessed by Fugl-Meyer Assessment (FMA), NIHSS, and Barthel Index scale (BI) to evaluate the severity of stroke and functional disability by trained and experienced neurologists before MRI examination. The FMA measurement consists of the motor function assessment items on upper limb and lower limb. BI is often used to evaluate the activity ability of daily living of stroke patient. For both of FMA and BI scales, a higher score reveals a better motor function or activity ability. On the contrary, a higher score of NIHSS, a comprehensive assessment to estimate the degree of neurological impairment, reflects a more serious stroke-related disability. Behavioral assessment and MRI examination were both conducted on the same day. To minimize operator-dependent bias, the neurologists were blinded to the patient grouping. The differences in clinical assessment scores between the RT and CT groups and before the first session and after the end of treatment were analysed by using the SPSS 22.0 software (Statistical Package for Social Sciences, Chicago, IL, USA). The results were considered significant at *p* < 0.05.

### 2.4. Resting Motor Threshold

For the rTMS treatment, the TMS coil was placed on the ipsilesional motor cortex. To determine the stimulation intensity, the resting motor threshold (RMT), defined as the minimal output of stimulation that could evoke muscle twitch of the contralateral first dorsal interosseous (FDI) or elicited a motor evoked potential (MEP) of at least an amplitude of 50 *μ*V in at least half of 10 consecutive stimuli recorded by electromyography [28], was determined for each patient by connecting the rTMS stimulator to an electromyogram apparatus (Dantec Keypoint System, Skovlunde, Denmark). The MEP signal was recorded from the surface Ag/AgCI electrodes placed on the FDI hand muscles.

### 2.5. rTMS Protocols

Patients in the RT group received 10 daily sessions of rTMS over the hand area of the ipsilesional primary motor cortex (M1) for a duration of 15 minutes using a Mag Pro butterfly-shape coil stimulator (MagVenture, Lucernemarken, Denmark). Each session of rTMS involved 30 trains of 50 pulses with 25-second intervals at 10 Hz and 90% RMT (total 1500 pulses/day). The stimulated motor cortex was determined and defined as the location that could elicit muscle twitch and the largest MEP of the contralateral FDI. As the ipsilesional hemisphere remained nonresponsive to TMS stimulation, the exact site of stimulation was defined as the location homologous to the contralesional motor cortex. Besides, during the rTMS treatment, the coil was positioned tangentially to the scalp of the stimulation target at a 45° angle from the midsagittal plane.

### 2.6. Acupuncture Strategy

Acupuncture was performed at the bilateral Fengchi acupoint and ipsilesional Baihui, Xuanzhong, Quchi, Hegu, Zusanli, and Sanyinjiao acupoints, with Baihui and Fengchi forward flat spines 0.5–1.5 inch, obliquely to the tip of the nose direction of the wind pool, and the remaining acupoints down to levels of 0.8–2.0 inches. The twisting angle was less than 90 degrees. The acupuncture treatment was performed by experienced and licensed acupuncturists. Each patient received thirty minutes of acupuncture treatment per day following rTMS treatment. Patients in both the RT and CT groups were also given antiplatelet aggregation drugs to improve blood circulation.

### 2.7. MRI Acquisition

All MRI data were acquired on a GE Signa HDxt 1.5 Tesla MR scanner (General Electric Medical System, Milwaukee, WI, USA) by using an 8-channel head coil. The DTI acquisition was performed by using a single-shot echo-planar imaging (EPI) sequence with the following parameters: TR/TE = 8500/96 ms, flip angle = 90°, field of view = 240 mm × 240 mm, matrix = 256 × 256, voxel sizes = 0.94 × 0.94 × 5.0 mm^3^, 32 axial slices with no gap, and acquisition time = 4 minutes and 50 seconds. The diffusion sensitive gradients were applied along 30 noncollinear directions with *b* value = 1000 s/mm^2^ to obtain the weighted images and one unweighted B0 image with *b* value = 0 s/mm^2^.

Along with the diffusion tensor imaging scan, high resolution anatomical T1-weighted images were also acquired for each subject using a three-dimensional-spoiled gradient recalled (3D-SPGR) sequence: TR/TE = 9.1/2.9 ms, flip angle = 20°, field of view = 240 mm × 240 mm, matrix = 256 × 256, voxel sizes = 0.94 × 0.94 × 1.2 mm^3^, and 124 slices with no gap.

### 2.8. Image Processing

SPM8 software (statistical parametric mapping, http://www.fil.ion.ucl.ac.uk/spm/) was used for preprocessing of DTI datasets to remove the head motion by aligning 30 diffusion weighted images to B0 image. Eddy current distortions were corrected by affine registration to the reference B0 image. Whole brain fiber tracking and reconstruction of the diffusion parameter images were evaluated in the DTI native space using interpolated streamline propagation algorithm with Diffusion Toolkit Software (http://trackvis.org/). Three eigenvalues (*λ*1, *λ*2, and *λ*3) and the maps of MD and FA were produced for each subject. During fiber tracking, if the FA value was lower than 0.2 or if the angle was less than 40°, the path tracking was stopped. All of the maps were normalized to the Montreal Neurological Institute (MNI) coordinate system and reinterpolated into isotropic voxels of 3.0 mm × 3.0 mm × 3.0 mm. Finally, to improve the signal-to-noise ratio of the maps, an isotropic Gaussian kernel (FWHM = 8 mm) was applied to complete the spatial smooth filtering. To detect the alteration of MD and FA values after the rTMS treatment, a paired *t*-test was adopted with a statistical significant level of *p* < 0.05. The two-sample *t*-test was also used to compare the images between the patients from RT and CT groups. To minimize the possible impact on the findings, age, gender, and duration of stroke were used as covariates in all statistical analyses.

### 2.9. Correlation Analysis

After comparing the FA maps between pre- and post-rTMS treatments, the motor-related brain regions, which revealed significant differences, were selected as regions of interest (ROI) to investigate the potential relationships between the alteration of diffusion parameters in these ROIs and clinical assessment improvement. Each ROI was defined as a cluster composed of 27 voxels around the peak coordinate of the difference area. For each ROI, the extracted values were manually checked and confirmed before the average value of the 27 voxels was obtained. Finally, Pearson correlation analysis was conducted between the diffusion parameters and clinical assessments including FM and BI to estimate their homogeneity.

## 3. Results

**Figure 1:**
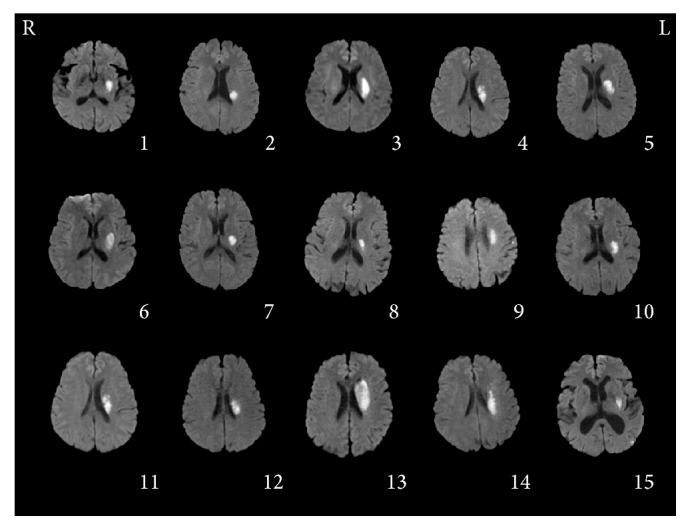
Individual diffusion weighted images in the axial view. The panel shows the slice with maximum infarct volume. Each subject is coded by the same serial number as the first row in Table 1.

Table 1 shows the demographic and clinical characteristics of the included stroke patients. No significant differences in age, gender, type of stroke, duration, or baseline behavioral scores were observed between the RT and CT groups at baseline.  Figure 1 illustrates the lesion location from the slice of maximum infarct volume on DWI images for each participant. All of the patients completed the 10 treatment sessions without reporting any adverse effects.

### 3.1. Behavioral Outcomes

Table 1 shows the descriptive pretreatment and posttreatment data for all clinical measures for both RT and CT groups. NIHSS, FMA, and BI scores showed significant changes after the treatment in both groups (NIHSS: RT: *p* = 0.003; CT: *p* = 0.001; FMA: RT: *p* = 0.001; CT: *p* = 0.026; BI: RT: *p* = 0.012, CT: *p* = 0.001). Additionally, the increase in FMA was significantly greater in the RT than in the CT group after the treatment (*p* = 0.041).

### 3.2. Factional Anisotropy Improvement

**Figure 2:**
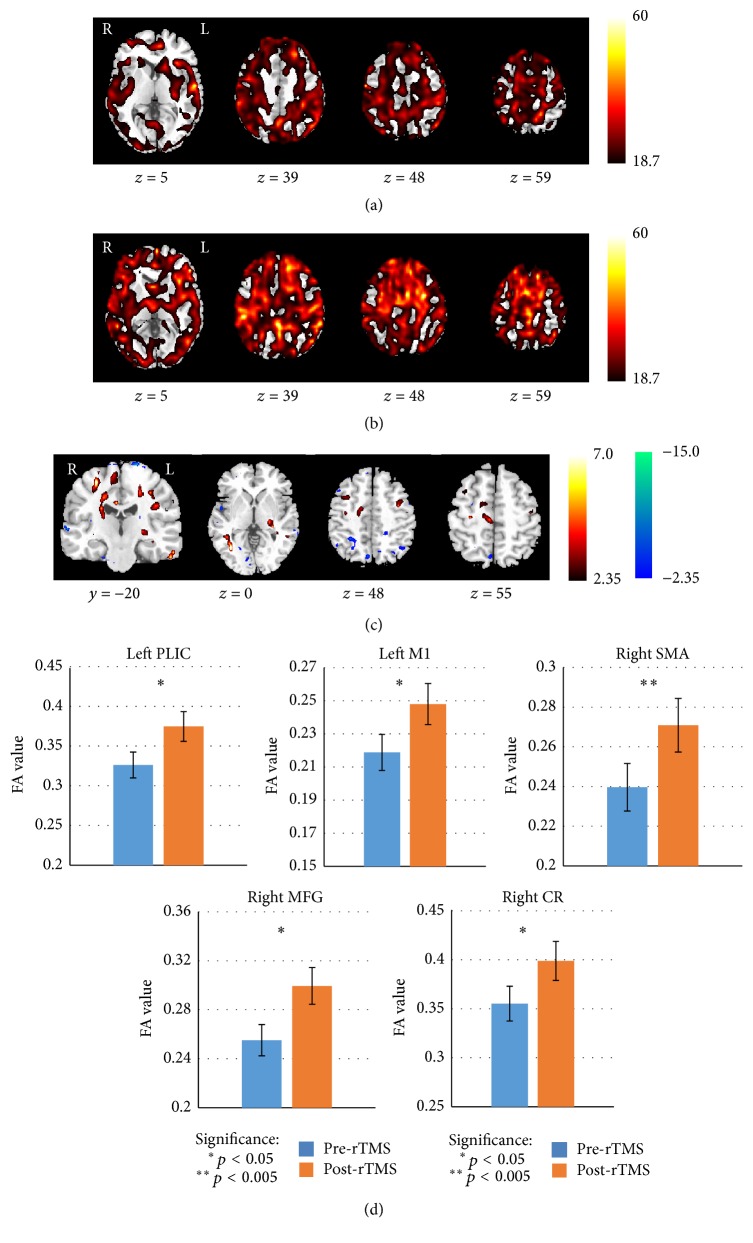
Comparison of FA maps between pre- and post-rTMS treatment for stroke patients in RT group. The FA results of one-sample *t*-test for stroke patients of pre- (a) and post-rTMS treatment (b). (c) Significantly changed brain areas are superimposed on the che2bet hemisphere of the Montreal Neurological Institute template brain in the three-view drawing (*p* < 0.05). The warm and cold tones separately indicate the increased and decreased FA value after rTMS treatment. (d) Bars represent the mean FA values. Vertical bars indicate estimated standard errors. Compared with the pre-rTMS treatment, the mean FA showed a significant increase after rTMS treatment in bilateral posterior limb of internal capsule (PLIC), left precentral gyrus (PG), right supplementary motor area (SMA), right middle frontal gyrus (MFG), and right corona radiate (CR).

**Figure 3:**
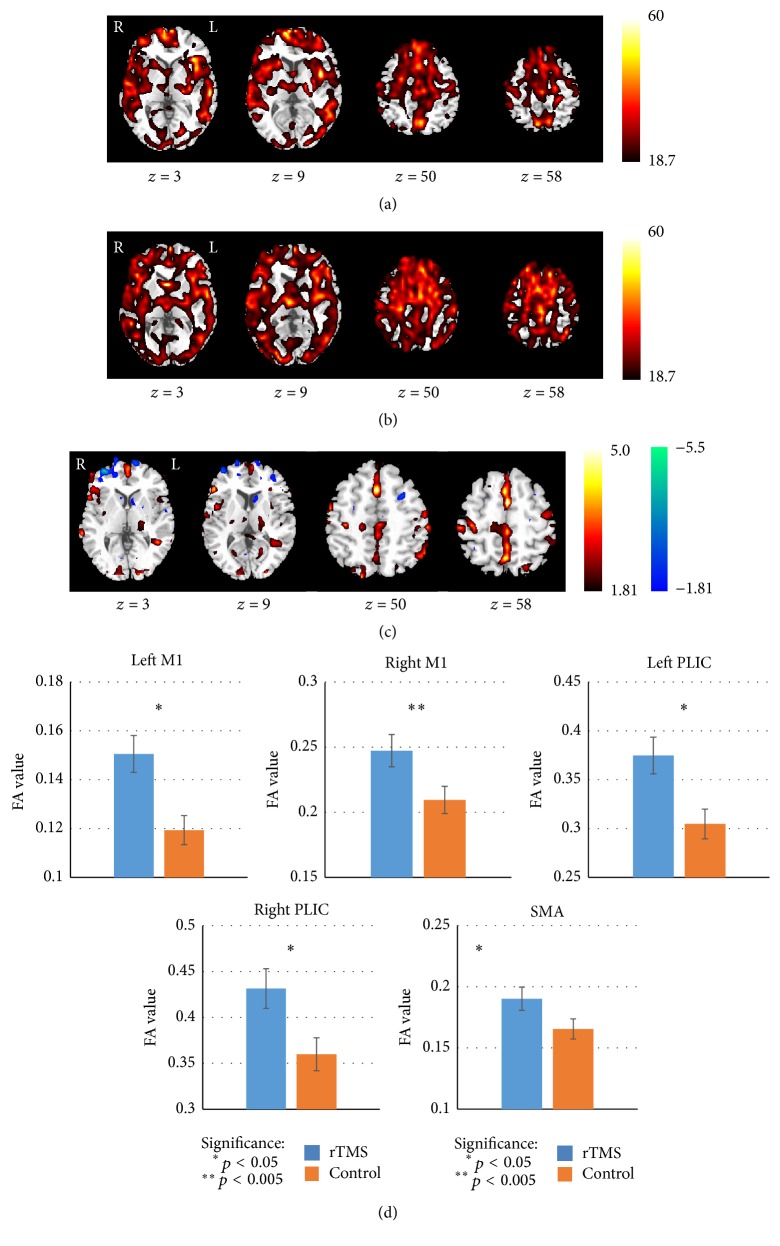
Comparison of FA maps between patients of RT and CT groups after treatment. The FA results of one-sample *t*-test for stroke patients of post-CT (a) and post-rTMS treatment (b). (c) Significantly changed brain areas are superimposed on the che2bet hemisphere of the Montreal Neurological Institute template brain in the three-view drawing (*p* < 0.05). The warm and cold tones separately indicate the increased and decreased FA value of RT. (d) Bars represent the mean FA values. Vertical bars indicate estimated standard errors. Compared with the CT, the mean FA value showed a significant increase after rTMS treatment in bilateral posterior limb of internal capsule (PLIC), primary motor area (M1), and supplementary motor area (SMA) in RT.

Paired comparisons between pre- and post-rTMS conditions revealed that the quantitative diffusion FA values of patients in the RT had increased significantly in the ipsilesional posterior limb of internal capsule (PLIC), M1, contralesional supplementary motor area (SMA), middle frontal gyrus (MFG), bilateral CST at the level of corona radiate (CR), and contralesional PLIC after 10 days of rTMS intervention (*p* < 0.05, uncorrected). The significant differences were demonstrated in Figure 2 and detailed information of significant clusters and peak voxels was recorded in Table 2. No significant changes were observed in the CT after a 10-day acupuncture and conventional medication treatment. Moreover, the quantitative diffusion FA value in the bilateral PLIC, M1, and SMA was significantly different between the RT and CT groups after rTMS and conventional treatment (*p* < 0.05, uncorrected) (Figure 3 and Table 3).

### 3.3. Relationship between Motor Improvement and White Microstructure

**Figure 4:**
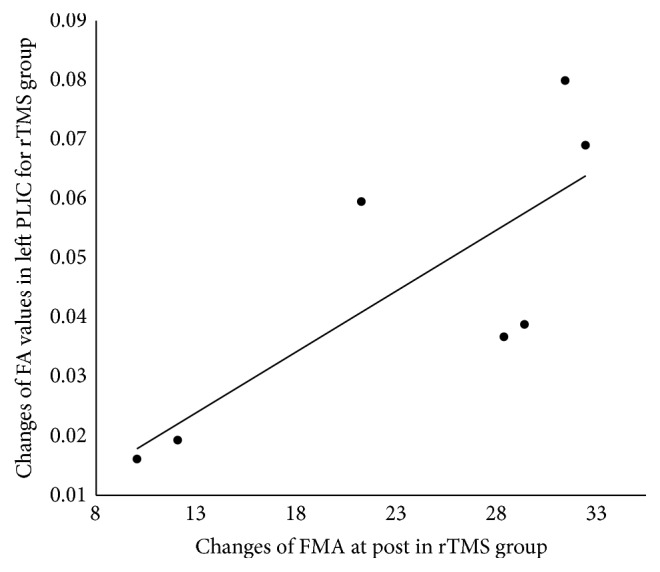
Relationship between changes of FA value in the ipsilesional posterior limb of internal capsule and changes of FMA score after rTMS treatment in the RT group (correlation coefficient *r* = 0.78, *p* = 0.039).

To evaluate the relationship between the quantitative FA values and the functional clinical recovery, we detected a significant and positive correlation between the altered FA value in the ipsilesional PLIC and changes in FMA scores in RT group (*r* = 0.78, *p* = 0.039) (Figure 4). For the CT group, although positive correlation was found, it was not significant (*r* = 0.27, *p* = 0.52). No significant correlation was observed in other areas.

## 4. Discussion

In this study, 10-day successive sessions of exciting high frequency (10 Hz) rTMS over the ipsilesional motor cortex were evaluated in acute ischemic stroke patients, as an add-on therapy to current therapy for its effect on motor function recovery. Voxel-based diffusion parameter analysis was applied to investigate the microstructural alteration after this treatment. The investigation showed that both RT plus CT (including acupuncture and medication) and CT alone improved the motor function of the affected limb. Furthermore, the improvement was greater in the RT + CT group than in the CT alone group as measured by FM score (Table 1). The significantly improved microstructural properties of motor-related white fibers and gray matter areas, which included the PLIC, M1, SMA, MFG, and CR, were also observed in the RT + CT group. The FA changes in the ipsilesional PLIC of rTMS-treated patients but not in that of control patients showed a linear relationship with the functional motor gains, suggesting a potential modulation of the motor function recovery and motor-related neural systems as well as the interhemispheric communication by repeated rTMS treatment in stroke patients. The results of this study also demonstrated that high frequency rTMS was a safe and tolerable add-on therapy for acute ischemic stroke patients.

Previous studies showed that rTMS alone was effective for motor recovery in stroke patients [14, 29–32]. In these studies, both single session [14, 29] and multiple sessions [30–32] of rTMS over affected hemisphere facilitated and enhanced motor recovery in stroke patients, in terms of movement accuracy [14], movement time [14], frequency of finger tapping [29], grip strength [30–32], and other motor aspects of motor function. However, so far, no study has reported FMA as a potential mechanism underlying high frequency rTMS-induced motor rehabilitation after stroke.

In this study, rTMS add-on therapy significantly increased FA values in the ipsilesional and contralesional PLIC and bilateral CR of stroke patients and this increase in the ipsilesional PLIC was positively correlated with the alteration of FMA scores. Although similar imaging results were observed in the CT group, they did not reach a significant level. These findings would reflect a boosting effect of rTMS on motor function recovery after stroke. Recent studies have shown that PLIC is mainly comprised of CST which control voluntary movement and is commonly used as the regions to the analysis of the integrity of CST [33, 34]. Compared to other brain regions, PLIC would have a closer relationship with motor function. In addition, studies on stroke patients observed that degree of damage to the PLIC (reflected as reductions in FA values) is correlated with poor motor function [21], and the regional FA changes of PLIC could predict changes of motor impairment [35]. Thus, an increased FMA score would represent improved motor ability, and the positive correlation may indicate that the bigger FA value increased the better motor recovery of the patients.

There is evidence that PLIC and CR were mainly comprised of CST which project from the motor cortex to the PLIC [34, 36, 37] and to brain stem [33]. Previous diffusion microstructural studies have showed significantly decreased FA values in ipsilesional [5, 38, 39] and contralesional [24, 40] PLIC and CST after stroke. The degree of FA reduction in PLIC was also correlated with the damage of motor ability [5, 24]. During rehabilitation, the FA value of both the affected [21, 41] and unaffected CST increased significantly [42], and it has been used to predict the long-term motor outcome after stroke [21, 43]. Furthermore, a 7-day HF-rTMS reduced the infarct volumes, improved glucose metabolism, and inhibited neuronal apoptosis in lesional area in mice with acute experimental stroke [44]. In our study, we placed high frequency rTMS stimulation over the ipsilesional M1 area which is the origin of CST. Therefore, the neuromodulation of rTMS is possibly associated with or modulated through this structural pathway that involved improved energy metabolism.

Except for the white matter structures, we also observed increased FA value in several motor-related gray matter areas including the bilateral M1, SMA, ipsilesional thalamus, paracentral lobule, and contralesional MFG after rTMS treatment. It is known that the brain motor network, consisting of the M1, SMA, and paracentral lobule [45, 46], is crucially involved in the voluntary motor control [47]. Although MFG is not part of the motor network, previous functional MRI studies illustrated that MFG may monitor and reinforce motor performance and cognitive and executive functions during stroke recovery [48–51]. Besides, thalamus is involved in the modulation of motor function [52].

The cortical interhemispheric competition theory suggests a dual facilitation and inhibition modulation mechanism of the excitability of motor cortex between bilateral hemispheres that may underlie the recovery of motor dysfunction [53]. Indeed, increased FA value in CST and the transcallosal M1-M1 tracts was reported during stroke rehabilitation [35, 38], supporting that both are associated with subsequent motor recovery. A recent study of low frequency rTMS (LF-rTMS) on contralesional hemisphere in stroke patients with motor dysfunction suggests that LF-rTMS could increase FA value of transcallosal motor fibers and modulate and assist adaptive neuroplastic changes in stroke patients [54]. Together with this study's findings of FA value increased in bilateral M1 and motor-related cortex, it can be inferred that these findings may reflect the neuromodulation and therapeutic effects of rTMS on microstructural plasticity in stroke patients.

There are several limitations in the present study. First, only a small number of patients were included in each group that reduced the analysis power in statistical analysis. Second, regarding the behavioral assessment, only FMA, NIHSS, and BI were used to evaluate rTMS-induced motor recovery. Because FMA is mainly for motor function assessment, whereas NIHSS and BI are too general to evaluate specific motor ability, more refined and targeted motor measurement could produce more detailed findings. Third, the potential effects of acupuncture treatment and medication received by both RT and CT groups cannot be excluded. In addition, the longer-term effects of rTMS treatment were not evaluated.

Although both RT and CT improved the neurological scores, motor function, and daily activity ability, a greater improvement in FMA scores was observed in RT group than in the CT group, suggesting a specific effect of HF-rTMS in this regard. A recent DTI study showed that a 4-week acupuncture treatment with conventional medication could improve the FMA score and white matter diffusion parameters compared to medication alone in stroke patients with unilateral motor deficits [55]. Thus, the lack of clear-cut different results between the RT + CT group and CT group in this study may have been obscured by the potential therapeutic effect of acupuncture present in both treatment groups, or the greater improvement in FMA score in the RT + CT group may still reflect additional beneficial effects of rTMS over CT on motor recovery. Future studies with separate controls of rTMS, acupuncture, and medication are needed to verify this possibility.

To our knowledge, this study is the first to have evaluated the effectiveness of high frequency rTMS add-on therapy in acute stroke patients with motor dysfunction using voxel-based diffusion parameter analysis. rTMS treatment plus CT for 10 days improved diffusion microstructures in motor-related white matter and gray matter brain regions that correlated with the behavioral recovery in stroke patients. Our results also suggest that change in FA values of the ipsilesional PLIC could be a potential biomarker for motor recovery in stroke patient that deserves further investigation.

## Figures and Tables

**Table 1:** Clinical data of included stroke patients.

Group	Number	Age (years)	Duration (days)	Gender	Lesion location	NIHSS		FM		BI	
						Pre	Post	Pre	Post	Pre	Post
RT	1	72	5	F	L_IC, BG	8	5	10	42	50	60
	2	63	5	M	L_IC, BG	6	4	13	34	60	70
	3	79	3	M	L_IC, BG	10	6	38	69	35	55
	4	64	6	F	L_IC, BG	11	3	77	89	25	70
	5	64	6	F	L_IC, BG	11	9	67	77	25	30
	6	74	3	F	L_IC, BG	6	3	32	60	65	80
	7	58	4	M	L_IC, BG	9	5	22	51	25	45
		67.71 ± 7.4	4.57 ± 1.27			8.71 ± 2.14	5.00 ± 2.08^*∗*^	37.00 ± 26.00	60.29 ± 19.54^*∗*△^	40.71 ± 17.42	58.57 ± 17.00^*∗*^

CT	8	56	4	F	L_IC	10	8	20	22	25	30
	9	76	3	M	L_IC, BG, CR	5	3	60	64	50	65
	10	53	6	F	L_IC, BG	9	7	14	35	40	55
	11	75	7	M	L_IC, BG	10	7	40	41	25	50
	12	61	5	F	L_IC, BG	9	6	22	27	30	40
	13	67	5	F	L_IC, BG, CR	7	3	63	69	80	90
	14	77	4	M	L_IC, BG, CR	11	5	11	18	20	30
	15	68	6	M	L_IC, BG	5	3	20	24	60	70
		66.63 ± 9.24	5.00 ± 1.30			8.25 ± 2.32	5.25 ± 2.05^*∗*^	31.25 ± 20.56	37.5 ± 19.37^*∗*^	41.25 ± 20.83	53.75 ± 20.83^*∗*^

BG: basal ganglia; BI: Barthel Index; CT: conventional treatment; CR: corona radiate; FM: Fugl-Meyer Assessment; L_IC: left internal capsule; NIHSS: National Institutes of Health Stroke Scale; RT: repetitive transcranial magnetic stimulation treatment; *∗* represents the significant difference between preclinical and postclinical scores (*p* < 0.05); △ represents the significant difference between the clinical scores of RT and CT groups.

**Table 2:** Brain regions with significant clusters and peak voxel coordinates showing FA difference between pre- and post-rTMS treatment.

Brain region	MNI coordinates (*x*, *y*, *z*)	*t* value	Voxel number
*Post > pre *			
Supp_motor_area_R	7, −23, 60	4.89	77
Frontal_mid_R	36, 10, 51	3.18	48
Precentral_L	−38, −2, 48	4.67	55
Precentral_R	27, −20, 54	4.22	73
Thalamus_L	−23, −23, 6	7.56	39
Temporal_inf_L	−59, −20, −30	6.89	31

*Post < pre*			
Precuneus_R	5, −68, 53	−4.74	32
Parietal_sup_R	27, −49, 51	−5.58	54
Angular_L	−44, −61, 48	−2.84	28

MNI: Montreal Neurological Institute; L: left; R: right.

**Table 3:** Brain regions with significant clusters and peak voxel coordinates showing FA difference between rTMS group and control group.

Brain region	MNI coordinates (*x*, *y*, *z*)	*t* value	Voxel number
*RT > CT*			
Supp_motor_area_L	0, −1, 61	7.91	294
Precentral_L	−44, −13, 60	3.63	75
Precentral_R	36, −23, 58	3.56	51
Postcentral_L	−48, −26, 54	2.78	59
Paracentral_lobule_L	−2, −31, 60	4.13	162
Precuneus_L	−2, −47, 60	3.9	372
Frontal_inf_tri_R	49, 32, 7	4.13	58
Thalamus_L	−23, −20, 6	2.68	51
Insula_R	45, 6, 9	2.96	46
Frontal_sup_medial_R	2, 59, 3	3.63	286
Temporal_mid_R	65, −36, 3	3.56	76
Occipital_sup_R	11, −96, 4	2.61	42

*RT < CT*			
Frontal_mid_R	29, 53, 3	−4.23	122
Caudate_L	−13, 17, 10	−2.39	58
Frontal_sup_L	−14, 67, 2	−2.76	50
Frontal_mid_L	−30, 10, 51	−3.1	29

MNI: Montreal Neurological Institute; L: left; R: right; RT: repetitive transcranial magnetic stimulation treatment; CT: conventional treatment.
